# The Use of an Air-Inflated Pillow for Assisted Recovery After General Anaesthesia in Horses: A Preliminary Study

**DOI:** 10.3390/ani15040564

**Published:** 2025-02-15

**Authors:** Zoë Vandaele, Charlotte Van den Broeke, Alexandre Merchiers, Stijn Schauvliege

**Affiliations:** Department of Large Animal Surgery, Anaesthesia and Orthopaedics, Faculty of Veterinary Medicine, Ghent University, 9820 Merelbeke, Belgium; zoe.vandaele@ugent.be (Z.V.); charlotte.vandenbroeke@ugent.be (C.V.d.B.); alexandre.merchiers@ugent.be (A.M.)

**Keywords:** anaesthesia, recovery, assisted, horses, quality, duration

## Abstract

Different assisted recovery techniques have been developed to enhance the safety and quality of equine recoveries. This case series evaluates the recovery of horses using an air-inflated pillow (Equi-lift^®^). With owner consent, the following data were collected from 19 horses undergoing open castration and 21 horses where a difficult recovery was anticipated: age, bodyweight, procedure, anaesthetic protocol, time required for proper positioning in the Equi-lift^®^, time to extubation, time to standing, number of attempts to stand, number of stimuli, behaviour during recumbency/first attempts, and overall impression. Horses were 1–24 years old, with a bodyweight of 483.5 ± 106.8 kg. The median time to properly position a horse into the system was 6 min (range: 1–15 min). The recovery duration was 42 min (range: 21–181 min), with a median of 4 attempts (range: 1–12 attempts) and 1 stimulus (range: 0–10) before fully standing. Recovery was calm in most horses (32/40) and moderate (with signs of anxiety and/or excitation) in 3/40 horses. In five horses, the use of this technique was discontinued. All horses recovered without any injuries or other immediate postoperative complications. In conclusion, this recovery technique shows promising results, but further studies are needed to confirm the findings.

## Simple Summary

The recovery phase is associated with many complications, such as fractures, luxations, etc. Despite advances in equine anaesthesia, the percentage of mortality and morbidity during this phase remains proportionally constant. Assisted recovery techniques have been developed to improve the safety and quality of recovery. The objective of this study was to describe the recovery when using a new technique, an air-inflated pillow (Equi-lift^®^, Waremme, Belgium). Because the horses are placed in a semi-standing position, it was hypothesized that it could reduce morbidity during recovery. Most horses showed a calm recovery; although, in some horses, the use of the system was discontinued due to different reasons. This indicates that a good case selection is essential when using this system. Further studies will be required to define these selection criteria. There were no direct complications observed during the use of this system.

## 1. Introduction

In equine anaesthesia, recovery is still a challenging phase, associated with complications, such as fractures, luxations, myopathies, or neuropathies [1]. Despite a reduction in mortality percentage [2] and advances in anaesthesia, the percentage of morbidity and mortality during recovery remains proportionally constant [3]. To minimize these risks, different recovery systems have been described. The most commonly used technique is head and tail rope assistance [4], which has been shown to improve recovery quality after short elective surgeries in comparison to unassisted recoveries [5] and after emergency exploratory laparotomy [6]. This is in contrast with another study [7], which did not show an improvement in the quality of recovery with head and tail-assisted recoveries, indicating the potential difficulties when studying these assisted techniques and demonstrating which technique is superior. Other recovery systems that have been described involve the use of a sling (e.g., use of the Anderson sling) [8,9], a tilt table [10], a water pool [11,12], a rapidly inflating–deflating air pillow system [13], or a recovery-enhancing device [14]. Each system has its own advantages and disadvantages.

Recently, a new recovery system has been developed in the form of an air-inflated pillow (Equi-lift^®^). Horses are placed on this pillow in a semi-standing position, which avoids the transition from lateral recumbency to standing. It is hypothesized that this technique will reduce the morbidities that could occur during the recovery phase. This preliminary study describes the recovery when using Equi-lift^®^ in 40 horses after general anaesthesia. The purpose of this study was to assess the feasibility and outcome of the use of this system, providing information about the recovery and its quality when using this type of recovery system.

## 2. Materials and Methods

### 2.1. Horses

Over a period of thirteen months (from October 2023 until November 2024), data were collected from horses that were presented for general anaesthesia and where the air-inflated pillow system was used (with owner consent).

Nineteen horses in this study were presented for open castration. They were identified as healthy patients after clinical examination and classified as an American Society of Anesthesiologists (ASA) status I. The other group consisted of horses that had a clear indication for the use of this recovery system, ranging from ASA status II-IV: fracture repair, expected difficult recovery (e.g., horses for computed tomography (CT) with a suspicion of Wobbler’s syndrome, older horses with osteoarthrosis, long anaesthesia with possible episodes of hypotension, and/or hypoxaemia, etc.), or wound repair with elevated risk of wound dehiscence during recovery. Based on previous experience with this system, the inclusion criteria were set based on weight and age; horses needed to weigh more than 350 kg and be older than 1 year.

### 2.2. Anaesthesia Protocol

Based on the history, reason for anaesthesia, and findings during the pre-anaesthetic examination, the attending anaesthetist selected the anaesthesia protocols. At the end of the anaesthesia, most horses received a bolus of sedation before being transported to the recovery box.

### 2.3. Equi-Lift^®^

**Figure 1:**
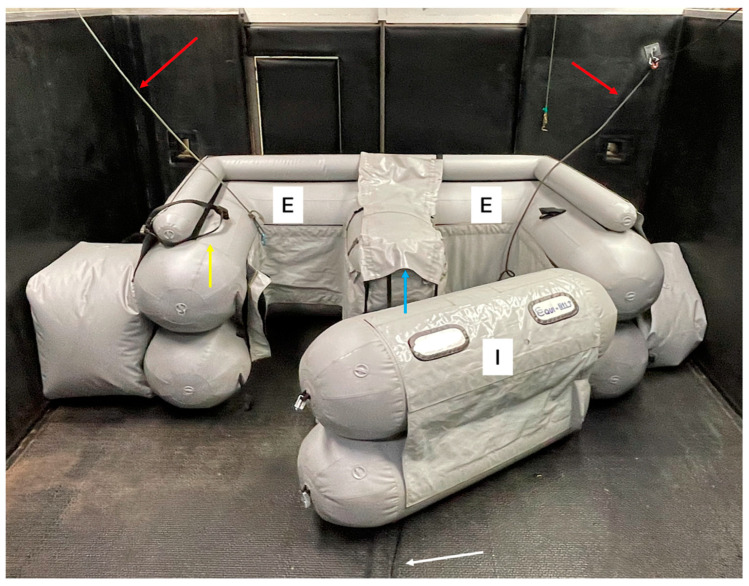
Equi-lift^®^ recovery system (partially opened) placed in a recovery box. E: part E of the pillow, I: part I of the pillow, red arrows: head and tail ropes, white arrow: tie-down straps to fix part I against part E during recovery, yellow arrow: neck collar, blue arrow: thoracoabdominal flap. Equi-lift ® (Warreme; Belgium) is the brand name of the air-inflated pillow.

The Equi-lift^®^ Model RC (Waremme; Belgium) is an air-inflated pillow designed to be used during recovery. It consists of two main parts. When looked at from above, one of these (E part) is shaped like a letter ‘E’. This is the main part on which the horse rests and consists of three inflatable layers (the lower two have a wide diameter, the uppermost a smaller one, see Figure 1). The second part (I part) is a part that closes the whole system and prevents the horse from falling out (see Figure 1). The dimensions of the Equi-lift^®^ are partially adapted to the size of the recovery box; the one in Figure 1 measures 300 cm long, 180 cm wide, and 110–130 cm high. The latter can be adjusted to the size of the horse (partial vs. full inflation with air). It is essential to always keep the lower layer of the E part fully inflated, to avoid any instability in the system. If there is variation in the size of the different recovery boxes in a hospital, as shown in Figure 1, two supplemental inflatable parts, in the shape of a cube, can be used to keep the pillow in place.

When placing the pillow inside the recovery box, it is important to have a head and tail rope available at the sides where the pillow is used (red arrows in Figure 1). After the placement of the pillow, it is essential to properly position the two tie-down straps (shown with a white arrow in Figure 1) clearly visible on the floor, in a position where they can easily be reached before the recovery. These straps are essential to retain part I against part E when the horse is recovering. A neck collar is also attached onto the pillow, on the side where the head will lie (shown by the yellow arrow in Figure 1).

This collar is placed around the neck of the horse and prevents it from lifting its forehand above the pillow. Finally, the flap that will be attached around the thorax and abdomen (shown with a blue arrow in Figure 1) should be opened, and the uppermost, small diameter layer of the E part deflated before transporting the horse into the recovery box.

### 2.4. Preparation for Recovery

In preparation for the recovery, both front legs were hobbled together to prevent the horse lifting one of its legs above the pillow. The same was carried out with the hind legs. If the horse had shoes, adhesive bandages were placed to avoid any damage to the pillow. At the end of anaesthesia, the horses were hoisted with hobbles on the legs (suspended upside down) and were transported into the recovery box.

### 2.5. Placement of the Horse in Equi-Lift^®^

The horse was positioned above the side wall of the E part (marked with the two letters ‘E’ in Figure 1), with the sagittal plane of the horse parallel to, and slightly outside, the midline of this side wall. The hoist was then lowered until the withers touched this part of the pillow. At this point, the tail rope was attached and pulled, so that it was under slight tension. At the same moment, the head rope was attached to the halter.

The hoist was then lowered until the horse was in a laterally recumbent position, mainly resting on the side wall of the E part, and the thoraco-abdominal flap of the pillow was attached around the horse. Following this, the hoist was lowered further, the hobbles, that were needed for transport, were removed, and the legs were manually lowered until the horse reached a semi-sternal position on the middle leg of the E part.

**Figure 2:**
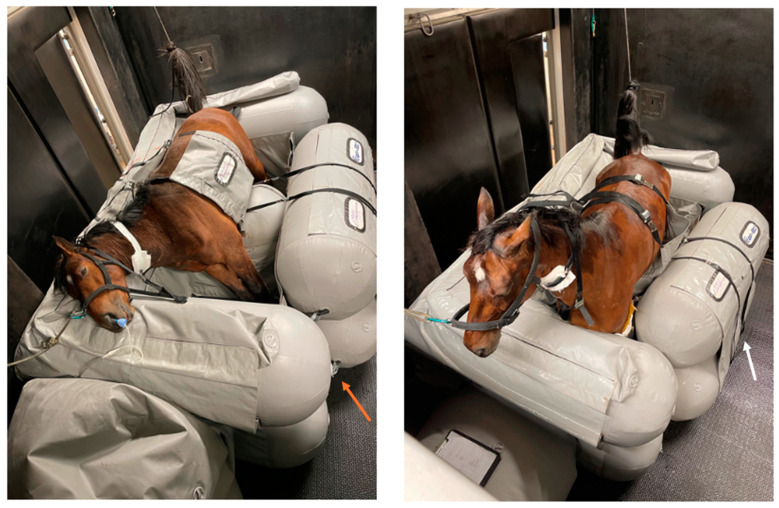
Equi-lift^®^ recovery system when a horse is installed in it (**left**), and when a horse is fully standing (**right**); white arrow: tie-down straps to fix part I against part E during recovery; orange arrow: straps cranial and caudal of the I part to fix it against part E.

The second part (I part) of the Equi-lift^®^ was then put into place to close the pillow and fixed to the E part using the two tie-down straps (white arrow in Figure 1 and Figure 2), as well as straps at the cranial and caudal side of the I part (orange arrow in Figure 2). At the same moment, the person that attached the halter to the head rope placed the neck collar around the neck. Finally, if needed in smaller horses, the upper part of the pillow was deflated until the feet touched the ground with the legs in slight flexion, and simultaneously the tension on the tail rope was checked and adjusted when needed. Once the horse was installed in the pillow, it received oxygen nasally at a rate of 15 L min^−1^.

Positioning the horse in the pillow is ideally carried out by 5 people. One person controls the hoist and ensures proper positioning. The second and third people, respectively, handle the head and the tail of the horse (help with positioning, attach head and tail ropes and neck collar), while the fourth and fifth people attach the thoraco-abdominal flap, detach the legs from the hoist, and close the I part of the pillow. Technically, it could be completed by three experienced users, where one person controls the positioning by using the hoist and gives instructions to the other persons, while another person controls the head (head rope and neck collar), and the last person attaches the tail rope and closes the thoracoabdominal flap. Finally, the last two people manually remove the hobbles and close the system, by attaching the I part to the E part by using the tie-down straps (see white arrows in Figure 2) and straps cranial and caudally (see orange arrow in Figure 2). For further illustration on the placement of a horse in the system, a video has been added (see Supplementary Materials). 

### 2.6. Recovery Scoring System

Data were collected during the recovery using a separate recovery scoring sheet (see Appendix A). The following objective data were collected: age, breed and bodyweight of the horse, type of procedure, anaesthetic protocol, time required to properly position the horse in Equi-lift^®^, time from the end of anaesthesia to extubation, time from end of anaesthesia to standing position, the number of attempts needed to stand, and the number of stimuli given.

Subjective descriptive data were also collected, including the behaviour of the horse during the recumbency and the first attempts, as well as the overall impression (calm behaviour/quiet, increased muscle tension, moderate recovery with slight excitation, or excitation/anxious). At the end of the recovery, a final score was given that described the recovery quality (calm, excitation, decided to stop the use of Equi-lift^®^).

## 3. Results

After placing the pillow into the recovery box, 15 min were required to fully inflate the pillow when using one electrical pump.

### 3.1. Horses and Anaesthesia Protocol

A total of 40 horses were recovered with Equi-lift^®^ (1–24 years old, mean ± SD (standard deviation), body weight of 483.5 kg ± 106.8 kg). Breeds that were presented were Warmblood horses (32/40), thoroughbreds (3/40), Arabian horses (2/40), a Belgian draft horse (1/40), an Appaloosa horse (1/40), and a Quarter horse (1/40).

A summary of the different types of procedures, the anaesthetic protocols, the durations of the anaesthesia, and the recovery and duration (if present) of hypotension and hypoxaemia during anaesthesia is shown in Table 1.

Most horses (37/40) were premedicated with acepromazine 20 µg kg^−1^ IM (Tranquinervin^®^, Dechra Veterinary Products, Lille, Belgium), usually followed by romifidine hydrochloride (Rominervin^®^, Dechra Veterinary Products, Lille, Belgium) 80 µg kg^−1^ IV (36/40) in combination with morphine 0,1 mg kg^−1^ IV (29/36), butorphanol (Torbugesic^®^, Zoetis, Zaventem, Belgium) 10 µg kg^−1^ IV (5/36), or methadone (Comfortan^®^, Dechra Veterinary Products, Lille, Belgium) 0.1 mg kg^−1^ IV (2/36). The other horses (4/40) received medetomidine hydrochloride 7 µg kg^−1^ IV (Domitor^®^, Orion Corporation, Espoo, Finland) in combination with either morphine 0.1 mg kg^−1^ IV (1/4) or methadone 0.1 mg kg^−1^ IV (3/4). After the placement of a 12-gauge catheter into the right or left jugular vein, non-steroidal anti-inflammatory medication and/or antibiotics were given pre-operatively.

In all horses, anaesthesia was induced with a combination of ketamine (Nimatek^®^, Dechra Veterinary Products, Lille, Belgium) 2.2 mg kg^−1^ IV and midazolam (Dormazolam^®^, Dechra Veterinary Products, Lille, Belgium) 0,06 mg kg^−1^ IV. Due to a human error, one horse received a dose of midazolam of 0.16 mg kg^−1^ instead of 0.06 mg kg^−1^ IV during the induction. Anaesthesia was maintained by TIVA (triple drip infusion) in 20 of the 40 cases (19 castrations and 1 CT) and with isoflurane (Isoflutek^®^, Laboratorios Karizoo, Barcelona, Spain) in an oxygen/air mixture in the other 20. Triple drip infusion consisted of ketamine 2000 mg, romifidine hydrochloride 25 mg (Rominervin^®^, Dechra Veterinary Products, Lille, Belgium), and guaiphenesin 25 g (Myorelax^®^, Dechra Veterinary Products, Lille, Belgium), diluted with 0.9% saline solution to a total volume of 500 mL. This was initially administered at 1–2 mL kg^−1^ h^−1^ and further titrated to effect. During inhalational maintenance (20/40), PIVA was administered with a CRI of romifidine hydrochloride at a rate of 40 µg kg^−1^ h^−1^ (14/20) or with medetomidine at a rate of 3.5 µg kg^−1^ h^−1^ (4/20). In the other two horses, anaesthesia was maintained with only isoflurane. Further details on the used anaesthetic protocols can be found in Appendix B.

In twenty-two horses, the trachea was intubated, where fifteen horses received mechanical ventilation, using the assisted or assisted-controlled ventilation mode; the other seven horses were breathing spontaneously.

For the horses that were presented for open castration (19/40), the duration of anaesthesia was not recorded but was estimated to be between 45 and 60 min. In the other horses, anaesthesia had a duration of 149 ± 78 min (mean ± SD). At the end of anaesthesia, most horses received a bolus of sedation (35/40), which consisted of 20 µg kg^−1^ romifidine IV (32/35) or 2 µg kg^−1^ medetomidine IV (3/35).

### 3.2. Data from Recovery

The median time needed to properly place the horse into the system was 6 min (range: 1–15 min; see Table 2). Once properly positioned, the median time to reach a standing position was 36 min (range: 13–176 min). This resulted in a median total time from the end of anaesthesia to a standing position of 42 min (range: 21–181 min; see Table 2). When looking at the horses that showed a calm (32/40) or moderate (3/40) recovery, these had a mean recovery time of 42 ± 13 min (mean ± SD); whereas, the horses where the use of the system was stopped, the total duration of the recovery was 124 ± 53 min (mean ± SD). The median time until the first attempt in the group of horses with a calm recovery was 26 min (range: 13–58 min). In the group of horses where the use of system was stopped, this time was 48 ± 13 min (mean ±min SD). In the group of horses that were orotracheally intubated for the procedure (22/40), the time until extubation was 16 ± 7 min (mean ± SD).

In all horses, the median number of attempts made before completely standing on all four legs was 4 (range: 1–12 attempts; see Table 2). Eight horses needed only one attempt to stand. Most of the attempts were calm and only partial, i.e., it was subjectively observed that horses tended to extend their limbs (forelimbs, hindlimbs, or both) and started to push themselves up, but as soon as they experienced some weakness or ataxia, they tended to cease their attempt and rest on the pillow for some additional time before making a new attempt. Some noise or tactile stimulation was regularly necessary for the horses to go to a fully standing position, but usually only 1 stimulus was needed (range: 0–10 stimuli). Stimuli were used when the horse had made several attempts without fully bearing weight on all four legs but remained calm, showing no further intent to try again, or when the recovery duration was subjectively deemed prolonged by the anaesthetist.

During recumbency in the Equi-lift^®^ (from the end of anaesthesia until the first attempts), the majority of the horses showed calm behaviour. During the first attempts to stand, most horses remained quiet; although, a few showed signs of anxiety and/or excitation. The final score of the recovery showed that 32/40 horses had a calm recovery, 1/40 had a moderate recovery with signs of anxiety, and 2/40 horses had a moderate recovery with signs of excitation. The two horses who showed signs of excitation received a second bolus of sedation, whereafter they recovered uneventfully.

In five cases, it was decided to stop using the Equi-lift^®^ by opening all air vents to rapidly deflate the pillow. These horses required 8 ± 4 attempts (mean ± SD) before fully standing. All five of these horses had received sedation prior to recovery, i.e., 20 µg kg^−1^ romifidine hydrochloride IV (3/5) or 2 µg kg^−1^ medetomidine IV (2/5). Four of these displayed signs of excitation during their first attempts, and an extra bolus of sedation with 20 µg kg^−1^ romifidine hydrochloride IV and/or acepromazine 20 µg kg^−1^ IM was administered. Further details can be found in Appendix C. These four horses continued to show excitation during their attempts but failed to fully stand. In two cases, after more than two hours of recovery, it was decided to stop the use of the system. These horses were further recovered with head and tail assistance, but one of these horses did not tolerate this and was finally recovered without assistance. Two other horses displayed aggressive attempts to stand 20–30 min into the recovery phase, with one horse repeatedly attempting to jump out of the system. Due to the risk of injuries, it was decided to stop the use of the Equi-lift in both. Assistance with head and tail ropes was attempted in both, but the horse that had tried to jump out of the system did not tolerate the ropes either and was recovered further without assistance. The fifth horse had a poor initial positioning into the system. After detaching of the horse, it was noticed that the hind quarter of the horse was partially tilted downwards due to insufficient tension on the tail rope. This horse made several attempts, but because of its poor position in the pillow, it was too difficult to properly push itself up to stand. After 82 min, it was decided to deflate the pillow and further recover the horse by head and tail assistance.

## 4. Discussion

Based on the findings of the current study, the use of the Equi-lift^®^ seems feasible. Indeed, despite the inclusion of 21 horses where a difficult or poor recovery quality was expected, all horses recovered without injuries, and most horses showed calm behaviour (32/40), with a median recovery duration of 42 min (range: 21–181 min) and a median of 4 attempts (range: 1–12 attempts) to stand. No direct complications were observed related to this type of recovery system (e.g., skin abrasions).

When comparing the recovery of horses with a calm recovery with that of horses where the use of the Equi-lift^®^ was discontinued, a longer recovery time (124 ± 53 min vs. 42 ± 13 min) with more attempts (8 ± 4 attempts vs. 4 attempts (range: 1–12)) and a somewhat longer time until the first attempt (48 ± 13 vs. 26 min (range: 13–58 min)) were observed in the latter group.

Different factors have been studied that influence the quality of the recovery from anaesthesia, such as hypoxaemia and a prolonged duration of anaesthesia [14]. When a difficult recovery is expected, e.g., fracture repair, long or emergency procedures, or whole limb cast [15], one of the available methods for assisted recovery is often used.

Based on questionnaires, 40–53% of clinics use an assisted technique during the recovery [16,17]. These methods have an influence on the quality and duration of the recovery [18], but there is limited scientific proof of which recovery system is superior. Indeed, the evaluation of the quality of recovery is inherently subjective [19,20], there are many confounding factors (which makes it difficult to compare cases to each other), and there is no clear consensus on a system to score recovery quality in horses, so under clinical circumstances, it is difficult to justify having a control group recovering without assistance in cases where this seems needed, etc.

For these reasons, the direct comparison of our findings with other studies is difficult. However, during the recovery phase, injuries such as fractures, luxations, or lacerations usually occur when the horse moves from lateral recumbency to a standing position. In Equi-lift^®^, the horses are immediately placed in a semi-standing position, possibly reducing the incidence of injuries that could occur due to persisting ataxia after general anaesthesia, especially in high-risk recoveries, such as fracture repairs.

The placement of the system into the recovery box occurred without problems. However, the installation of the pillow and the recovery process require more time and additional personnel. The installation of the pillow should be completed by at least two people, and full inflation of the pillow takes 15 min when using one inflating pump. This time could be shortened if using two electrical pumps at the same time.

During the course of the study, the time needed to properly position the horse into the system became shorter, illustrating a learning curve when using this system frequently.

The total duration of the recovery phase using the Equi-lift^®^ was 42 min (range: 21–181 min). This time is comparable with head and tail rope assistance reported in Arndt et al. (2019; [5]) and in Rüegg et al. (2016; [7]) and with the rapidly deflating pillow reported in Ray-Miller et al. (2006; [13]). Horses that recovered in the Equi-lift^®^ needed 4 attempts (range: 1–12 attempts) before fully standing on their four legs, where eight horses needed only 1 attempt. Although the number of attempts is similar to the findings with head and tail rope assistance in Arndt et al. (2019; [5]), it should be noted that the majority of horses in the current study tended to make a modest attempt, gently trying to push themselves upwards, but as soon as they experienced some difficulty, they tended to rest on the pillow for a while longer before making a new attempt. Retrospectively, it could be argued that the number of attempts in the current study is an overestimation, because minor attempts, where the horse only partially lifted its front or hind quarter, should have been ignored. Subjectively, in the authors’ opinion, the use of the pillow may reduce the risk compared to head and tail rope assistance, where horses can fall violently during an unsuccessful attempt.

In the group of horses that recovered calmly, part E of the Equi-lift^®^ was deflated partially in eight cases after positioning. These were small horses where partial deflation was required to allow the horses to stand completely on four legs. Importantly, the lowest layer of part E should never be deflated during recovery, as this reduces the stability of the system. Further observations were two horses that showed signs of anxiety when fully standing but not during the previous phases of recovery. Three horses were clearly leaning onto the system after a few attempts of trying to stand, and four horses were subjectively in a poor position to stand after a few attempts. These horses had either moved sideways (towards part I of the system, resulting in less support underneath the thorax/abdomen) or forwards or backwards (i.e., sliding down and sinking through the forehand or hindquarters). To avoid this, it is important to firmly attach the thoracoabdominal flap and use the head and tail ropes to keep the horse in place. In the authors’ opinion, the thoracoabdominal flap in future models should be positioned somewhat further away from the I part compared to the current model. However, after stimulation, all four of these horses recovered uneventfully.

During the recovery of five horses, it was decided to stop the use of the Equi-lift^®^. This shows that a good case selection before the use of this system is important, but further studies will be needed to clearly identify which factors these will be. An advantage of this system is that it can be rapidly deflated in case of emergency, and horses can be further recovered by head and tail rope assistance, without the need to anesthetize the horses again. The pillow can be deflated quickly by opening all air vents; within 5 min, it is fully deflated. In three cases, the pillow could be removed quite easily after deflation; in two other cases, it was left in place for the safety of the personnel. In the authors’ opinion, if it can be carried out safely, it is preferable to remove the pillow to minimize the risk of the horse tripping over its components.

After data collection, the mortality of the horses that were recovered with this system was reviewed. In total, 3 out of the 40 horses were euthanized later due to a poor prognosis, related to their initial presenting condition. Two horses showed signs of moderate to severe ataxia and were euthanized due to a diagnosis of hemangiosarcoma with secondary compressive myelopathy or seizure-like episodes that could not be controlled medically. One horse was euthanized due to severe tenosynovitis of digital flexor tendon sheath with adhesions and lesions in the flexor tendons. It is noteworthy that, despite the severity of the lesions in all these horses, only one of them showed a poor recovery.

### 4.1. Limitations of the Equi-Lift^®^

A limitation of this novel recovery system is that, in its current form, it is less suitable for smaller horses. Indeed, previous experience of the authors with this system showed that, due to the relatively large space between the three ‘legs’ of part E, it is more difficult to achieve a stable position for smaller horses. Especially when they wake up and start to move, there is a larger risk that they move cranially or caudally and gradually slide off the middle ‘leg’ of part E. Both the height at the withers and the length of the horse from shoulder to tail are important, but because body weight is easier to measure, one of the criteria in the current study was that only horses weighing more than 350 kg were included. The authors estimate that the height at the withers should be at least 150 cm. Most large horses seem to fit into the system, including the Belgian draft horse of 805 kg.

Due to the nervous character and strong flight response of horses, some horses may show signs of anxiety or excitation when recovering in this system. Based on this, one of the exclusion criteria in the present study was an age of less than 1 year. Indeed, younger horses are not used to being handled, so any assisted recovery system could lead to excitation. Although the character of the horse was not used as an exclusion criterion, it may also be hypothesized that it is contra-indicated to use the Equi-lift^®^ on very nervous or unhandled horses. Indeed, one of the horses where it was decided to stop the use of Equi-lift^®^ because of signs of excitation, was a very nervous horse before premedication. Nonetheless, it must be noted that the vast majority of horses in this study, with varying breeds and characters included, tolerated the use of the pillow surprisingly well.

Finally, it may be argued that the use of the hobbles on the front and hind legs during the recovery phase poses a risk for excitation. The use of such hobbles is recommended by the manufacturer to prevent horses lifting an individual leg above (and over) a wall of the pillow. In the current study, sufficient distance was allowed between both the front and hind legs, and all horses seemed to tolerate the use of the hobbles well.

### 4.2. Limitations of Study Design

This study is limited by the absence of a control group and a relatively small sample size. As such, it should be considered a case series that includes a diverse range of procedures, anaesthesia protocols, horse breeds, temperaments, and ages. However, additional research is required to draw conclusions about recovery quality or safety compared to unassisted or alternative assisted recovery techniques and to substantiate the promising findings. Horses that are presented for exploratory laparotomy due to colic usually show poorer quality of recovery due to the long duration of the procedure, peri-anaesthetic complications, such as hypoxemia and hypotension, etc. [21]. Nonetheless, the system was not used in colic cases in this study, due to concerns about potential risks, such as increased abdominal pressure and traction or friction on the surgical incision caused by the pillow.

It could be hypothesized that this type of recovery system could improve oxygenation during recovery, since less atelectasis would be expected in a semi-standing position compared to lateral recumbency. Further studies should be performed to prove this statement. An adaptation of the system should be considered, to ensure it also fits ponies or smaller horses. Further research will be needed to provide information on recovery with this adaptation.

## 5. Conclusions

Despite being more labour- and time-intensive, we can conclude that the air-inflated pillow, Equi-lift^®^, seems like a promising way to recover horses after general anaesthesia, especially those with a high-risk recovery, but further research is needed to demonstrate its safety in larger numbers of horses, define exclusion criteria, and compare the recovery quality to other techniques described in the literature.

## Figures and Tables

**Table 1:** Results (type of procedure, premedication, maintenance, duration of anaesthesia, duration of hypotension if present, duration of hypoxemia if present, duration of recovery, and number of attempts) of horses that showed calm and good recovery quality, moderate recovery quality, and excitation with discontinuation of the use of Equi-lift^®^.

Procedure	Nr. of Cases	Age (Years; Range)	Premedication	Maintenance	Duration of Anaesthesia	Duration of Hypotension	Duration of Hypoxaemia	Duration of Recovery	Nr. of Attempts (Range; Median)
Calm, good recovery quality									
Open castration	19	1–14	A, RMo	Triple drip	45–60 min	Unknown	Unknown	19–75 min	1–10 (3)
Ulna fracture	2	6–16	A, MMe	Isoflurane +/− medetomidine CRI +/− ketamine CRI	215–275 min	15–20 min	N/A	39–51 min	7–10 (9)
CT or MRI	4	9–16	A, RMo, RB	Triple drip, isoflurane +/− romifidine CRI	40–135 min	15 min (1/5)	N/A	33–60 min	1–3 (2)
Wound repair	2	4–18	A, RMo	Isoflurane + romifidine CRI	70–105 min	N/A	N/A	35–37 min	5–7 (6)
Cardiac procedures	2	6–12	RB, RMe, RMo	Isoflurane + romifidine CRI	180–193 min	N/A	N/A	47–67 min	3–7 (4)
Dental procedure	1	19	RMo	Isoflurane + romifidine CRI	77 min	N/A	N/A	53 min	8
Mandibular fracture	1	24	A, RMo	Isoflurane + romifidine CRI	190 min	N/A	N/A	42 min	2
Cast change	1	3	A, RMo	Isoflurane + romifidine CRI	165 min	15–20 min	N/A	24 min	1
Total	32								
Moderate recovery quality									
CT	1	20	A, RB	Triple drip	30 min	N/A	N/A	21 min	3
Cardiac procedure	1	7	RB	Isoflurane + romifidine CRI	175 min	N/A	N/A	45 min	10
Wound repair	1	1	RMo	Isoflurane + romifidine CRI	165 min	N/A	N/A	47 min	10
Total	3								
Excitation, discontinued use of Equi-lift ®									
Arthroscopy	1	8	A, RMo	Isoflurane + romifidine CRI	140 min	20–25 min	20–30 min	55 min	12
Cervical stenosis	1	2	A, MMo	Isoflurane + medetomidine CRI + ketamine CRI	195 min	25–30 min	N/A	181 min	9
Ulna fracture	1	1	A, RMe	Isoflurane + romifidine CRI	275 min	N/A	N/A	145 min	7
Cardiac procedure	1	7	RMo	Isoflurane + romifidine CRI	50 min	N/A	N/A	86 min	10
Arthrodesis PIP joint	1	13	A, MMe	Isoflurane + medetomidine CRI	195 min	N/A	N/A	80 min	3
Total	5								

A: acepromazine, RMo: romifidine hydrochloride with morphine, RMe: romifidine hydrochloride with methadone, RB: romifidine hydrochloride with butorphanol, MMe: medetomidine with methadone, MMo: medetomidine with morphine. Hypotension was defined as mean arterial pressure below than 70 mmHg, and hypoxaemia as arterial partial pressure below 80 mmHg for at least 15 min.

**Table 2:** Results from the data collection (subjective and objective data) during recovery when using Equi-lift^®^.

Objective Data		Subjective Data	
Time to position horse into system	Median: 6 min (range: 1–15 min)	Behaviour during recumbency	Calm: 39/40 Increased muscle tension: 2/40
Time from positioning the horse to standing	Median: 36 min (range: 13–176 min)	Behaviour during first attempts	Quiet: 30/40 Anxious: 5/40 Excitation: 5/40
Time from end of anaesthesia to standing	Median: 42 min (range: 21–181 min)	Overall impression	Quiet: 32/40 Moderate recovery: 3/40 Excitation: 5/40
Number of attempts	Median: 4 attempts (range: 1–12 attempts)	Final score min	Calm recovery: 32/40 Moderate recovery: 3/40 ⇒ With signs of anxiety: 1/40 ⇒ With signs of excitation: 2/40 Decided to stop use of Equi-lift^®^: 5/40
Number of stimuli	Median: 1 stimulus (range: 0–10 stimuli)		

## Data Availability

Data are contained within the article.

## Supplementary Materials

The following supporting information can be downloaded at https://www.mdpi.com/article/10.3390/ani15040564/s1, Video S1: The installation of an air-inflated pillow for recovery of horses.

## Appendix B. Details on Anaesthetic Protocols Used

**Premedication**	**Dosage**	**Number of Horses**
Acepromazine	20 µg kg^−1^ IM	37/40
Romifidine hydrochloride - With morphine - With methadone - With butorphanol	80–120 µg kg^−1^ IV	36/40
	0.1–0.2 mg kg^−1^ IV	29/36
	0.1 mg kg^−1^ IV	2/36
	10 µg kg^−1^ IV	5/26
Medetomidine hydrochloride - With morphine - With methadone	7–8 µg kg^−1^ IV	4/40
	0.1 mg kg^−1^ IV	1/4
	0.1 mg kg^−1^ IV	3/4
**Induction**	**Dosage**	**Number of horses**
Ketamine	2.2 mg kg^−1^ IV	40/40
+ midazolam	0.06 mg kg^−1^ IV	40/40
**Maintenance**		**Number of horses**
TIVA (triple drip infusion ketamine, guaifenesin, romifidine)		20/40
Isoflurane in oxygen/air mixture		20/40
+ CRI romifidine	40 µg kg^−1^ h^−1^	14/20
+ CRI medetomidine	3.5 µg kg^−1^ h^−1^	4/40
**Sedation before recovery**		**Number of horses**
No sedation given		5/40
Sedation given:		35/40
Romifdine hydrochloride	20 µg kg^−1^ IV	32/35
Medetomidine	2 µg kg^−1^ IV	3/35

## Appendix C. Details on Horses That Were Unfit for Recovery System

**Type of surgery**	Arthroscopy multiple joints	1/5	
	Cervical stenosis	1/5	
	Ulna fracture	1/5	
	TVEC	1/5	
	Arthrodesis PIP	1/5	
**Duration of anaesthesia**	171 ± 83 min (mean ± SD)		
**Duration of recovery**	124 ± 53 min (mean ± SD)		
**Number of attempts**	8 ± 4 attempts (mean ± SD)		
**Sedation before recovery**	5/5	Romifidine hydrochloride	3/5
		20 µg kg^−1^ IV	
		Medetomidine 1.5–2 µg kg^−1^ IV	2/5
**During recovery**	4/5	Romifidine hydrochloride	2/5
		20 µg kg^−1^ IV	
		Acepromazine 20 µg kg^−1^ IM	1/5
**Hypotension**	Yes (2/5)	Duration: 20–30 min Treatment: fluid therapy, dobutamine CRI	
**Hypoxaemia**	N/A	N/A	
